# Editorial: Innovative use of imaging techniques within plant science

**DOI:** 10.3389/fpls.2022.1079022

**Published:** 2022-11-22

**Authors:** Gregorio Egea, Alexander Bucksch, Lisbeth G. Thygesen

**Affiliations:** ^1^ Area of Agroforestry Engineering, School of Agricultural Engineering, University of Seville, Seville, Spain; ^2^ Department of Plant Biology, University of Georgia, Athens, GA, United States; ^3^ Warnell School of Forestry and Natural Resources, University of Georgia, Athens, GA, United States; ^4^ Institute of Bioinformatics, University of Georgia, Athens, GA, United States; ^5^ Department of Geosciences and Natural Resource Management, University of Copenhagen, Frederiksberg, Denmark

**Keywords:** imaging, non-destructive measurements, unmanned aerial vehicle (UAV), microscopy, microspectroscopy, artificial intelligence

Several examples in the history of biology show how technological advances have facilitated fundamental discoveries in biology. The development and application of imaging techniques in plant sciences represent such an example that is currently unfolding. By using image analysis, spatially resolved information can be obtained that allows new questions in the field to be explored. Furthermore, when applied for example in crop monitoring, quality control or management, these techniques allow objective real-time decisions to be made, often based on non-destructive measurements and a reduction in time and labor that could also translate into cost savings.

This Research Topic brings together research papers that demonstrate how image-based techniques can help solve actual problems in the world of plant sciences. Generally, the presented papers offer image-based solutions to assess plant disease status, predict and detect grain and fruit yield, and analyze wood samples for their species and quality. These general application areas were achieved with a range of imaging instruments from the microscopy level to airborne image collection with unmanned aerial vehicles (UAV).

Zhang et al. (2021) tackle the long-standing and laborious yield prediction problem to precisely quantify yellowness in canola flowers. In doing so, they propose a UAV method to effectively estimate yield in Canola (Brassica napus L.) from airborne imagery. Their remote-sensing solution is to define a normalized yellowness vegetation index (NDYI) that demonstrated high predictive performance for seed yield.

Using similar technology, Shi et al. (2022) propose the use of UAV-based multispectral imagery and machine learning (ML) models for aboveground biomass (AGB) and leaf area index (LAI) estimation of two intercropping species (mung bean and red bean) in tea plantations. Five ML algorithms were evaluated based on the vegetation indices derived from the UAV multispectral images as well as the actual AGB and LAI data. Their results show that two models (Support Vector Machine and Back Propagation Neural Network) outperformed the AGB and LAI prediction of red bean and mung bean as compared to other ML models.

Crop disease detection using image-based techniques is also a field that experiences growth due to the positive impact crop productivity and greater environmental and economic sustainability of agriculture. In this sense, Jiang et al. (2022) have conducted a study aimed at assessing the severity of wheat stripe rust using a low-cost approach based to evaluate images of infected leafs obtained by smartphones. This approach may represent a compromise between the sometimes-subjective visual disease assessment and symptoms assessment using costly devices such as multi- and hyper- spectral cameras. Along, Leiva et al. (2022) compared the performance of two low-cost image-based methods for predicting Fusarium Head Blight (FHB) infection in winter wheat seeds. The two analysis methods use RGB images of wheat seeds to provide various morphological traits of the seed, which were used to predict FHB using multiple regression models.

The development of robots for automatic fruit harvesting is a growing discipline due to the increasing costs of manual harvesting and the difficulty of finding skilled labor. Accurate and robust detection of fruits under natural conditions is crucial for the success of automatic fruit harvesting with robots. In this line of work, Hou et al. (2022) have developed a methodology based on the use of binocular cameras and deep learning to improve both citrus fruit detection and 3D localization under natural lighting conditions in commercial citrus orchards. To this end, an improved version of the YOLO v5s model is proposed for citrus detection, Cr-Cb chromatic mapping together with Otsu threshold algorithm and morphology processing are used to extract citrus shape, and a geometric image model for 3D citrus localization. Liu et al. (2022) present another work aimed at improving the automatic detection of fruits under natural conditions using deep learning models. In their case study, the authors have developed an anchor-free detector based on the CenterNet architecture that outperforms other tomato detection methods.

Another innovative application of the use of image-based techniques is that developed by Husaini et al. (2022) for the detection of fraudulent saffron. Saffron adulteration is a major problem, because Saffron is an expensive spice that is normally used as hand-picked dried flower stigmas. As a technological advancement, the authors have successfully tested two new methods for detecting adulterated saffron, one based on the use of a low-cost optical microscope (Foldscope) in combination with a chemical staining technique for visual identification of fake saffron samples, and another based on deep learning to automatically classify images taken with Foldscope and a smartphone.

Berger et al. (2021) report on a study in which image data obtained using darkfield and fluorescence microscopy was used to quantify the histology in cross sections of whole maize stems. This information was used for phenotyping different maize lines. The method developed makes it possible to assess unusually large cross sections, i.e., in the cm range. It is possible to quantify plant anatomy and autofluorescence after excitation with ultraviolet and/or visible light.

Determining the wood species or genus of timber and wooden artefacts based on light microscopy is important when controlling wood trade, especially to protect endangered tree species. However, wood identification is a skill that requires training and expertise, which means that far less wood is controlled than one could wish for from a conservation viewpoint. Adding to the challenge is the limited availability of microscopy images from known species in species-rich forests. Lopes et al. (2022) describe an exciting first step towards addressing this problem. Their approach involves neural networks to generate artificial images based on microscopy images of known species. In a second step, the method increases the number of images available per species to train neural networks to be able to identify the wood species in microscopy images of unknown species.

The article by Ponzecchi et al. (2022) describes a study where chemically modified wood was studied using Raman micro-spectroscopy. The novelty of this article lies in the development and test of a miniature climate chamber that makes it possible to adjust the relative humidity of microtomed sample sections mounted below a normal coverslip while they are presented to the instrument. In addition to the advantage of securing a well-defined and adjustable relative humidity, the setup has the advantage of being compatible with immersion objectives.

Together, the articles of this Research Topic illustrate the many useful applications that are currently being explored within this active field of research and development.

## Author contributions

All authors listed above have made substantial contributions to the work. All authors have approved the final manuscript for publication.

## Acknowledgments

We thank all authors and reviewers for their contributions to this Research Topic and for the support of the editorial office.

## Conflict of interest

The authors declare that the research was conducted in the absence of any commercial or financial relationships that could be construed as a potential conflict of interest.

## Publisher’s note

All claims expressed in this article are solely those of the authors and do not necessarily represent those of their affiliated organizations, or those of the publisher, the editors and the reviewers. Any product that may be evaluated in this article, or claim that may be made by its manufacturer, is not guaranteed or endorsed by the publisher.

